# Emergency Cesarean Section in a Dichorionic Diamniotic Twin Pregnancy With Hydrops Fetalis: A Report of a Critical Case

**DOI:** 10.7759/cureus.56207

**Published:** 2024-03-15

**Authors:** Shahzad Ahmad, Sagar Karotkar, Revat J Meshram, Sham Lohiya, Aditi Rawat

**Affiliations:** 1 Department of Paediatrics, Jawaharlal Nehru Medical College, Datta Meghe Institute of Higher Education & Research, Wardha, IND; 2 Department of Neonatalogy, Jawaharlal Nehru Medical College, Datta Meghe Institute of Higher Education & Research, Wardha, IND

**Keywords:** multidisciplinary care, neonatal resuscitation, advanced maternal age, emergency cesarean section, hydrops fetalis, dichorionic diamniotic twins

## Abstract

This case report describes the emergent scenario of a 41-year-old primipara at 31.2 weeks of gestation, presenting with abdominal and back pain in the context of a dichorionic diamniotic twin pregnancy complicated by hydrops fetalis. The patient, with a history of hypertension, hyperthyroidism, and a cervical stitch in place, underwent an emergency lower segment cesarean section. The ultrasound revealed an intrauterine left footling in one twin, contributing to the suspected hydrops fetalis. Neonatal complications arose, particularly with Baby B, necessitating immediate resuscitation and intensive care. Successful outcomes were achieved through a well-coordinated multidisciplinary approach involving obstetricians, neonatologists, and anesthesiologists. This case underscores the importance of prompt recognition, timely interventions, and collaborative care in managing complex pregnancies, shedding light on the challenges associated with dichorionic diamniotic twin pregnancies and emphasizing the need for ongoing research to refine perinatal strategies.

## Introduction

Multiple gestations, particularly dichorionic diamniotic twin pregnancies, present unique challenges and complexities in obstetric care. The incidence of twin pregnancies has been on the rise, attributed in part to advancements in assisted reproductive technologies [1]. While these pregnancies often result in favorable outcomes, they also carry an increased risk of complications, necessitating vigilant antenatal monitoring and timely interventions [2]. Advanced maternal age is a significant factor associated with twin pregnancies, as seen in the presented case. A maternal age of 35 years or older is linked to an elevated risk of adverse perinatal outcomes, including preterm birth and chromosomal abnormalities [3]. In this context, the case of a 41-year-old primipara with a dichorionic diamniotic twin pregnancy underscores the importance of thorough antenatal care and risk assessment in older gravidas.

The presence of maternal comorbidities such as hypertension and hyperthyroidism adds a layer of complexity to the management of twin pregnancies. Hypertensive disorders complicate approximately 6-8% of pregnancies and are associated with an increased risk of preterm birth, fetal growth restriction, and other adverse outcomes [4]. Similarly, maternal hyperthyroidism can impact fetal well-being and contribute to obstetric complications, emphasizing the need for meticulous monitoring and intervention [5]. The utilization of cervical stitches in pregnancies with a history of cervical incompetence aims to prevent preterm delivery and reduce the risk of adverse neonatal outcomes [5]. However, the effectiveness of cervical cerclage in improving perinatal outcomes remains a subject of ongoing research and debate [6].

Hydrops fetalis, a rare condition characterized by abnormal fluid accumulation in fetal compartments, further complicates the presented case. While hydrops fetalis can have various etiologies, its occurrence in a dichorionic diamniotic twin pregnancy is uncommon. This condition is associated with a high perinatal mortality rate, emphasizing the need for prompt diagnosis and intervention [7]. The case also highlights the significance of neonatal resuscitation and intensive care in emergent situations. Immediate recognition of fetal distress during delivery and swift neonatal intervention are crucial in mitigating potential complications and improving overall outcomes [8].

## Case presentation

**Figure 1:**
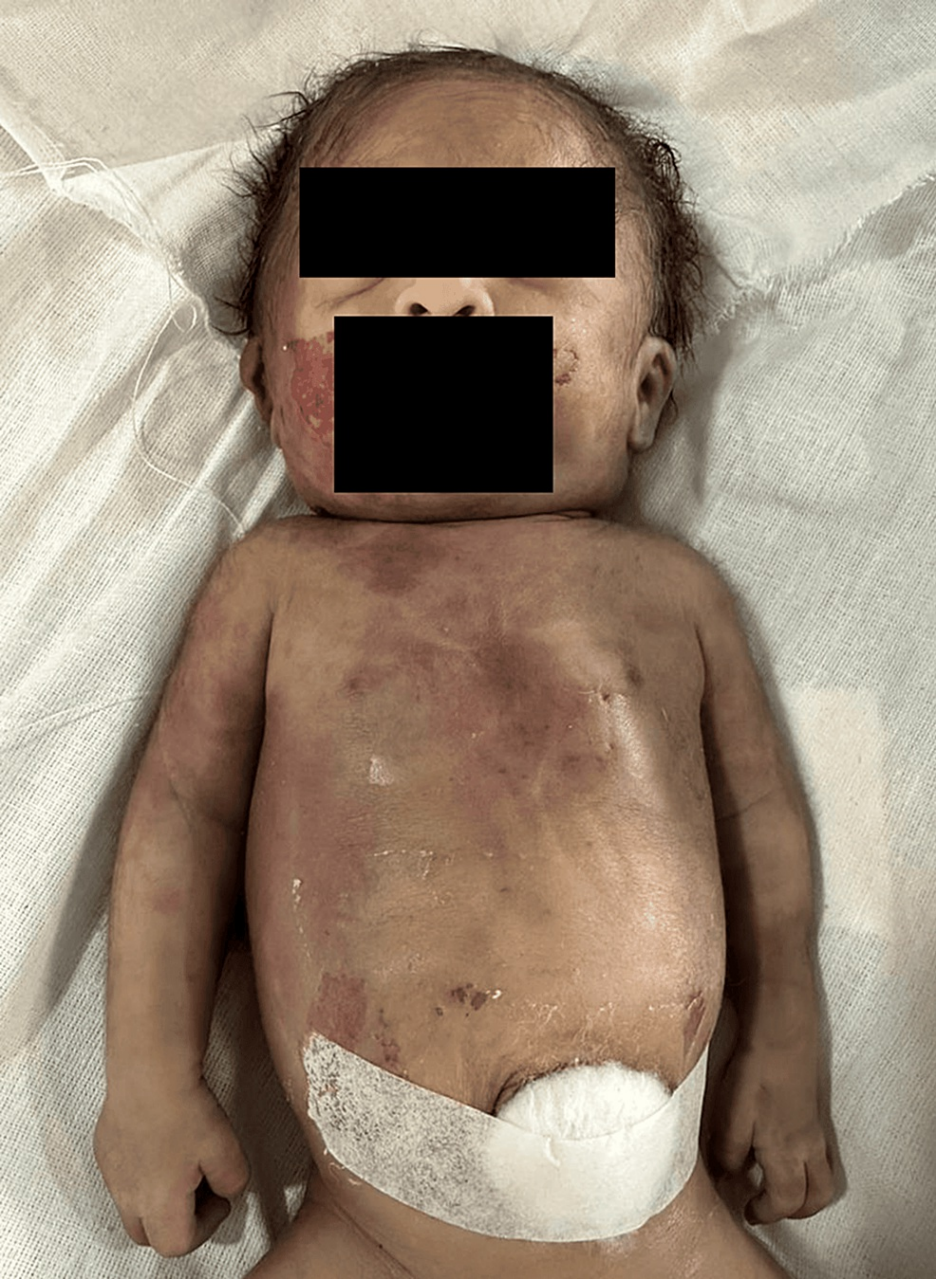
Subcutaneous edema noted over the scalp, neck, and abdomen, suggesting a possibility of hydrops fetalis

A 41-year-old female presented to the emergency department of a tertiary care hospital with complaints of abdominal and back pain. Upon gathering her medical history, her husband explained that she was a primipara at 31.2 weeks of gestation, carrying twins. She also had a known case of hypertension and hyperthyroidism, with a cervical stitch in place. The ultrasound report revealed a dichorionic diamniotic twin pregnancy with intrauterine left footling (IULF) F2 (maternal left) carrying a weight of 1,662 grams. Subcutaneous edema was noted over the scalp, neck, and abdomen, suggesting a possibility of hydrops fetalis (Figure 1).

A physical examination confirmed generalized edema throughout her body. In response, the doctor promptly transferred her to the operating theater for an emergency lower segment cesarean section. The presentation of the babies showed that they were in the breech position. The pediatrician received the babies, and suctioning was performed. Baby A was in good health and was placed in a warmer. However, Baby B, a 1.4 kg male child, did not cry immediately after birth and experienced bradycardia. Consequently, he was swiftly transferred to the neonatal intensive care unit. After assessing the vital signs, the necessary treatments were initiated. The pediatrician explained the child’s condition to the father, obtained consent, and started treatment, including intravenous administration.

**Figure 2:**
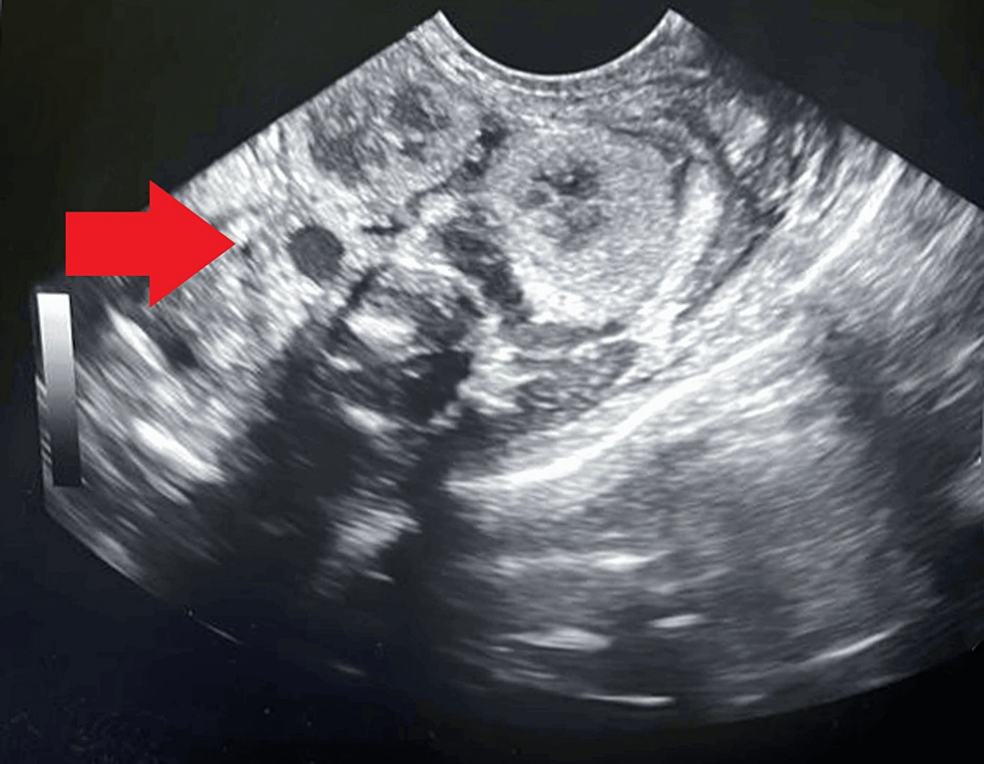
Ultrasonography revealed right-crossed renal ectopia and Grade I renal parenchymal disease with minimal ascites

As Baby B continued to experience bradycardia, anesthesiologists were briefed upon their arrival. The baby was incubated with an endotracheal tube (size 3, fixed at 7.5). Cardiopulmonary resuscitation was performed, and after 30 seconds, the baby was successfully revived. He was connected to a ventilator in pressure support ventilation mode, with positive end-expiratory pressure set at 5, peak continuous airway pressure at 10, and fraction of inspired oxygen at 80%. Pharmacological management included adrenaline, milrinone, sodium bicarbonate, and antibiotics (intravenous injections of ampicillin and gentamicin). Close monitoring was advised for the baby, and despite being on ventilator support, oxygen saturation was maintained. Further ultrasonography revealed right-crossed renal ectopia and Grade I renal parenchymal disease with minimal ascites (Figure 2).

The baby was observed and started on more potent antibiotics. The endotracheal tube was removed on the seventh day, and oxygen was administered via an O2 hood. The baby maintained oxygen saturation, with the hood used intermittently for monitoring. The pediatrician decided to shift the baby to the mother’s side while continuing the administration of antibiotics until the completion of the prescribed course.

## Discussion

The presented case underscores the challenges and critical decisions associated with emergency cesarean sections in dichorionic diamniotic twin pregnancies complicated by hydrops fetalis. Several factors contributed to the complexity of this case, including the patient’s advanced maternal age, underlying hypertensive disorder, hyperthyroidism, and the presence of a cervical stitch. Advanced maternal age has been associated with increased risks during pregnancy, including preterm birth and chromosomal abnormalities [9]. In this case, the patient’s age of 41 may have contributed to the complexity of the pregnancy. Additionally, the presence of hypertension and hyperthyroidism necessitated careful monitoring throughout the gestational period. The cervical stitch, indicating a history of cervical incompetence, further heightened the risk for preterm delivery [10].

Hydrops fetalis is a rare but severe condition characterized by abnormal fluid accumulation in fetal compartments. In dichorionic diamniotic twin pregnancies, it is even less common. The etiology can vary, including immune and non-immune causes, with potential consequences for both fetuses [7]. In this case, the IULF observed in one twin may have contributed to the compromised fetal circulation, leading to the development of hydrops fetalis. The immediate neonatal care provided to Baby B was crucial in addressing post-delivery complications. Bradycardia and the need for resuscitation necessitated a rapid transfer to the neonatal intensive care unit. Endotracheal intubation, ventilator support, and pharmacological interventions were pivotal in stabilizing the infant. The successful outcome in this case aligns with the importance of prompt and comprehensive neonatal care in improving outcomes for infants with perinatal complications [11].

The identification of right-crossed renal ectopia and Grade I renal parenchymal disease in Baby B highlights the importance of thorough postnatal evaluation. Such anomalies may be associated with intrauterine factors or may be incidental findings. The successful management of the renal anomalies and the subsequent weaning off of ventilator support indicate the resilience of neonates when provided with appropriate and timely medical interventions [12]. The successful management of this critical case emphasizes the importance of a multidisciplinary approach involving obstetricians, neonatologists, anesthesiologists, and other allied health professionals. Timely communication, collaborative decision-making, and swift intervention contributed to the positive outcome for both the mother and the neonates.

## Conclusions

The case highlights the crucial need for comprehensive and multidisciplinary care, particularly in pregnancies involving older mothers and additional medical conditions such as hypertension and hyperthyroidism. The swift decision for a cesarean section in response to the observed hydrops fetalis emphasizes the critical role of timely interventions in addressing potential life-threatening situations during pregnancy. The successful neonatal resuscitation and subsequent intensive care for Baby B illustrate the effectiveness of a well-coordinated neonatal team in managing complications. Furthermore, identifying and managing renal anomalies emphasizes the importance of postnatal evaluations and collaborative efforts among various medical specialties. Overall, this case contributes valuable insights into the evolving field of obstetrics, emphasizing the significance of continuous research and a multidisciplinary approach in optimizing outcomes for mothers and neonates in complex perinatal scenarios.
